# Evidence of a Recessively Inherited CCN3 Mutation as a Rare Cause of Early-Onset Parkinsonism

**DOI:** 10.3389/fneur.2020.00331

**Published:** 2020-05-15

**Authors:** Steven R. Bentley, Suliman Khan, Marco Öchsner, Susitha Premarathne, Zain Aslam, Javed Y. Fowdar, Jamila Iqbal, Muhammad Naeem, Christopher A. Love, Stephen A. Wood, George D. Mellick, Alex M. Sykes

**Affiliations:** ^1^Griffith Institute for Drug Discovery, Griffith University, Nathan, QLD, Australia; ^2^Department of Biotechnology, Quaid-i-Azam University, Islamabad, Pakistan

**Keywords:** parkinson, genetic, CCN3, NOV, early-onset, extracellular matrix

## Abstract

The study of consanguineous families has provided novel insights into genetic causes of monogenic parkinsonism. Here, we present a family from the rural Khyber Pakhtunkhwa province, Pakistan, where three siblings were diagnosed with early-onset parkinsonism. Homozygosity mapping of two affected siblings and three unaffected family members identified two candidate autozygous loci segregating with disease, 8q24.12-8q24.13 and 9q31.2-q33.1. Whole-exome sequence analysis identified a single rare homozygous missense sequence variant within this region, *CCN3* p.D82G. Although unaffected family members were heterozygous for this putative causal mutation, it was absent in 3,222 non-Parkinson's disease (PD) subjects of Pakistani heritage. Screening of 353 Australian PD cases, including 104 early-onset cases and 57 probands from multi-incident families, also did not identify additional carriers. Overexpression of wild-type and the variant CCN3 constructs in HEK293T cells identified an impaired section of the variant protein, alluding to potential mechanisms for disease. Further, qPCR analysis complemented previous microarray data suggesting mRNA expression of *CCN3* was downregulated in unrelated sporadic PD cases when compared to unaffected subjects. These data indicate a role for CCN3 in parkinsonism, both in this family as well as sporadic PD cases; however, the specific mechanisms require further investigation. Additionally, further screening of the rural community where the family resided is warranted to assess the local frequency of the variant. Overall, this study highlights the value of investigating underrepresented and isolated affected families for novel putative parkinsonism genes.

## Introduction

In most cases, the cause of Parkinson's disease (PD) is unknown, and although the risk may be influenced by a number of common genetic (1) and environmental factors (2), only a minority of cases can be ascribed to known genetic causes. These rare monogenic causes of parkinsonism often arise in and are detected through the study of consanguineous families from remote regions. Notably, mutations in *DJ-1* (3), *PINK1* (4, 5), *DNAJC6* (6), *SYNJ1* (7), *PLA2G6* (8), and the putative genes *PODXL* (9) and *ADORA1* (10) were identified in consanguineous parkinsonism families. While most PD cases do not possess these rare genetic variants, their discovery provides insight into the cellular mechanisms involved in the development of the disease, and ongoing screening of affected families is a powerful platform to identify further candidates. To this end, a consanguineous family from a rural district in the Khyber Pakhtunkhwa province, Pakistan, who presented with early-onset parkinsonism but had screened negative for known causes of recessive parkinsonism, underwent further genetic analysis. This study reports the finding of a rare, putatively pathogenic, p.D82G mutation in *CCN3* (also known as *NOV*) in this family. The gene encodes a secreted matricellular protein, which may have a role in adhesion, cellular signaling, cell migration, angiogenesis, and calcium homeostasis (11).

## Materials and Methods

### Genetic Analysis

Due to the remote location of the family, only the proband (IV:3) was available for diagnosis by an expert movement disorder specialist. Details of the family were acquired through interviews with the proband. DNA was extracted from venous blood donated by patients from the Khyber Pakhtunkhwa province, Pakistan (Ethics: Ref. 65/IRBEB/PGMI/LRH), using the phenol chloroform method, and from Queensland, Australia (Ethics: Ref. ESK/04/11/HREC), using a salting-out method described previously (12).

Approximately 300,000 single-nucleotide polymorphisms (SNPs) were genotyped using the HumanCytoSNP-12 BeadChip and the iScan system (Illumina) in the two patients IV:3 and IV:5, as well as three unaffected members, III:2, III:5, and IV:4. All samples had SNP call rates >95%. Homozygosity mapping was performed using GenomeStudio (Illumina) and Homozygosity Mapper (13). Copy number variation (CNV) detection was performed using the cnvPartition plugin within GenomeStudio (Illumina).

Whole-exome sequencing (WES) was performed in patient IV:3 using the Nextera Rapid Capture Exome Enrichment chemistry and sequenced under the 2 ×75-bp pair-end configuration on the MiSeq sequencer (Illumina) at the Griffith University DNA sequencing facility. The sample produced an average read depth of 30 × over the 45-Mb target region, with ~93.5% of calls above Q30. The data were prepared as recommended by the Genome Analysis Toolkit (GATK) developers (14). Sequence variants differing to the human consensus sequence hg19/GRCh37 were identified by the HaplotypeCaller algorithm (GATK) and annotated by ANNOVAR (15). Variants were filtered by the following parameters: (1) missense variants; (2) gnomAD minor allele frequency (MAF) <0.001; (3) homozygous.

The mutation was confirmed by Sanger sequencing, which was performed by amplifying the region surrounding the mutation using the following primers: 5′-GGTTTCTCCTTGTCTCGCCT-3′ (forward) and 5′-GCTGCAGGAGAAGAGGTCAA-3′ (reverse). Amplification products then underwent BigDye Terminating v3.1 reaction which were analyzed on the genetic analyzer 3130 × l (Applied Biosystems) at the Griffith University DNA sequencing facility.

### High-Resolution Melt Analysis

High-resolution melt (HRM) analysis was used in 320 Australian PD samples to assess genetic variants in the 135-bp region surrounding the putative mutation, encompassing 57% of exon 2. HRM primers were 5'-GCTCATGCTGTCTGGTGTGT-3′ (forward) and 5′-GATTACCGTGCAGATGCCA-3′ (reverse). Briefly, products were amplified using the GoTaq kit (Promega) with 1.5 mM MgCl_2_, 200 μM dNTPs (Bioline), 200 nM primers (Sigma-Aldrich), and 1 μM dsDNA binding Syto9 dye, with the following PCR profile on a RotorGene 6000 (QIAGEN Inc.): 95°C for 2 min followed by 40 cycles of 95°C for 15 s, 60°C for 15 s, and 72°C for 15 s. Fluorescence was acquired at the 72°C step, and the product underwent high-resolution melt between 83° and 93°C.

### Quantitative PCR

Quantitative PCR (qPCR) was used to verify observations from microarray data of human olfactory neurosphere-derived (hONS) cells (16), available at www.ebi.ac.uk/arrayexpress (Accession: E-TABM-724). Total RNA was extracted from hONS cells donated by nine unrelated idiopathic Queensland PD cases and eight unaffected controls using TRIzol (Thermo Fisher Scientific) at 60–80% confluence. RNA (200 ng) was converted to cDNA using the SuperscriptIII First-Strand Synthesis SuperMix kit (Thermo Fisher Scientific). qPCR analysis amplified *CCN3* as well as endogenous controls *RPL13* and *TBP*, using the following primers: *CCN3* forward 5′-CGGCGGTAGAGGGAGATAAC-3′, *CCN3* reverse 5′-GCCTGTAAGCTGCAAGGGTA-3′, *RPL13* forward 5′-CCTGGAGGAGAAGAGGAAAGAGA-3′, *RPL13* reverse 5′-TTGAGGACCTCTGTGTATTTGTCAA-3′, *TBP* forward 5′-CCACTCACAGACTCTCACAAC-3′, and *TBP* reverse 5′-CTGCGGTACAATCCCAGAACT-3′. Products were amplified using the PowerUp SYBR green kit (Applied Biosystems), the thermal cycling conditions were UDG activation 50°C for 2 min, polymerase activation 95°C for 2 min, followed by 40 cycles of 95°C for 15 s, 60°C for 15 s, and 72°C for 1 min. A relative standard curve was used to determine the expression of *CCN3* to the geometric mean of *RPL13* and *TBP*. Statistical analysis was conducted in R (v3.5.1). Briefly, two outliers (controls) were removed for high values [relative expression (log2): 2.3 and 1.7]. Normality and Levene's test of equal variance were assessed using the ggplot2 (v3.0) and car (v3.0-5) packages. A Student's *t*-test was conducted under the assumptions of normal distribution and equal variance using the stats (v3.5.1) package. The Bonferroni correction was used to control for multiple comparisons.

### V5-Tagged Expression Construct Design

Briefly, cDNA was prepared from a control hONS cell line and the *CCN3* coding sequence, lacking the stop codon, was amplified using the Pfusion HF polymerase (New England Biolabs). Next, the *CCN3* amplicon was inserted into pDONR201 and then into the pEF-DEST51 expression vector using the Gateway BP and LR Clonase II Enzyme mix, respectively (Thermo Fisher Scientific). The c.A245G (NM_002514) point mutation was introduced by the QuikChange Lightning Multi Site-Directed Mutagenesis Kit (Agilent Technologies) using the following primers: 5′-CTGGAGCCATGCGGCGAGAGCAGTGGC-3′ (forward) and 5′-GCCACTGCTCTCGCCGCATGGCTCCAG-3′ (reverse). Correct sequence identity was verified by Sanger sequencing.

### Cell Culture and Transfection

HEK293T cells were cultured in Dulbecco's modified Eagle's medium (DMEM; Thermo Fisher Scientific) supplemented with 10% fetal calf serum (FCS; Thermo Fisher Scientific) at 37°C in a humidified atmosphere with 5% CO_2_. For immunoblot experiments, 75,000 cells were plated into a 24-well plate overnight (Nunc™) and transiently transfected using Lipofectamine 2000 (Thermo Fisher Scientific). For immunostaining experiments, cells were plated at a density of 37,500 cells overnight on poly-ornithine (Sigma-Aldrich)-coated glass coverslips. The cells were either fixed or lysed 48 h post-transfection, and supernatants were also collected by centrifugation for 10 min at 300 × g to remove cell debris and analyzed. Lysis was performed using 10 mM Tris, 150 mM NaCl, 1 mM EDTA, and 1% Triton-X100, incubated for 15 min on ice followed by centrifugation for 5 min at 4°C. Fixation was achieved by incubating coverslips in 4% paraformaldehyde (PFA) for 10 min.

### Immunoblotting

Immunoblots were performed using Tris-Glycine gels and standard sodium dodecyl sulfate–polyacrylamide gel electrophoresis (SDS-PAGE) protocols. Nitrocellulose membranes were probed with rabbit-anti-V5 (1:3,000; Cell Signaling Technology) and mouse-anti-α-tubulin (1:18,000; Sigma-Aldrich) antibodies overnight at 4°C, followed by goat-anti-mouse-680RD and goat-anti-rabbit-800CW secondary antibodies (both 1:24,000 LI-COR) for 60 min at room temperature. Membranes were imaged on an Odyssey-Fc imaging system (LI-COR).

### Immunostaining

Coverslips were permeabilized and blocked in PBS containing 10% horse serum and 0.3% Triton-X100 for 60 min. CCN3 was detected using rabbit-anti-V5 antibody (1:1,000) for 90 min at room temperature and detected using donkey-anti-rabbit-555 secondary antibody (1:1,000, Thermo Fisher Scientific). Coverslips were counterstained with 4′,6-diamidino-2-phenylindole (DAPI; Sigma-Aldrich) and imaged on an Olympus FV1000 confocal microscope.

## Results

**Figure 1:**
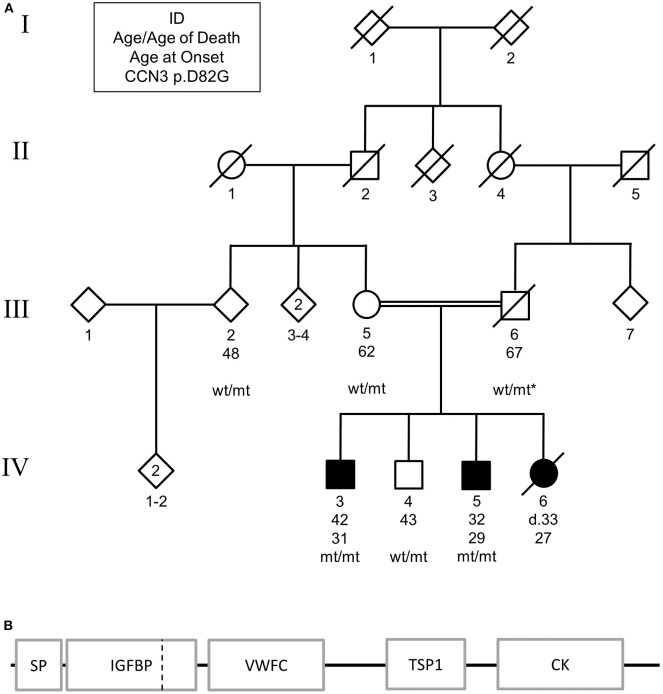
**(A)** Pedigree of family carrying *CCN3* p.D82G homozygous mutation. Solid shapes indicate those affected by parkinsonism. Squares represent males, circles represent females, and diamonds represent undefined. * indicates an inferred genotype. Non-essential pedigree information has been omitted or modified to protect the privacy of the family. **(B)** Schematic of mutation location in protein structure. SP, signal peptide; IGFBP, insulin-like growth factor binding protein; VWFC, von Willebrand factor type C; TSP1, thrombospondin type-1 repeat; CK, cysteine-knot, C-terminus. Dashed vertical line represents mutation location.

Patient IV:3 was diagnosed with levodopa-responsive parkinsonism at the age of 31 after developing rigidity and gait abnormalities. The symptomatology progressed, and at the age of 36 presented with severe rigidity, frequent falling events, mild tremor, hypomimia, difficulty swallowing, and a stooped posture. Magnetic resonance imaging (MRI) scans were unremarkable (data not shown). The proband had reported patient's IV:5 and IV:6 also presented with similar symptomatologies, while the parents were not affected (Figure 1A). CNV analysis and WES in IV:3 excluded known causes of genetic parkinsonism, including *PARK2* and *SNCA* dosage. Homozygosity mapping of the two affected siblings suggested that two autozygous loci, 8q24.12-8q24.13 and 9q31.2-q33.1, segregated with disease. Collectively, these encompassed 97 protein-encoding genes.

WES identified one missense variant in these genes with a MAF <0.001, *CCN3* p.D82G (exon 2, c.A245G, NM_002514) on chromosome 8 using the parameters described. Subsequent Sanger sequencing showed that the affected sibling (IV:5) was also homozygous for this mutation, while the unaffected brother (IV:4), mother (III:5), second-degree relative (III:2) were found to be heterozygous for this mutation (Figure 1A). By inference, the father (III:6) would also be heterozygous for this mutation. The variant resided in the insulin-like growth factor binding domain of the CCN3 protein (Figure 1B).

The variant was rare, with a MAF of 4.012 ×10^−6^ in the gnomAD dataset, and was not identified in the exomes of 168 Pakistani subjects from the Greater Middle East (GME) Variome Project (17); 3,222 subjects of Pakistani heritage (18); or 3,044 subjects from the AnnEx database, which contained a mixture of ethnic groups and movement disorders, including those with parkinsonism as the predominant phenotype (https://annex.can.ubc.ca). Further, HRM analysis and previous WES data did not identify the mutation in 353 Australian PD samples, including 104 early-onset cases (age at onset <50 years) and 57 probands from multi-incident families. The variant had a CADD score of 24.7 (19), which was suggestive of a deleterious variant.

**Figure 2:**
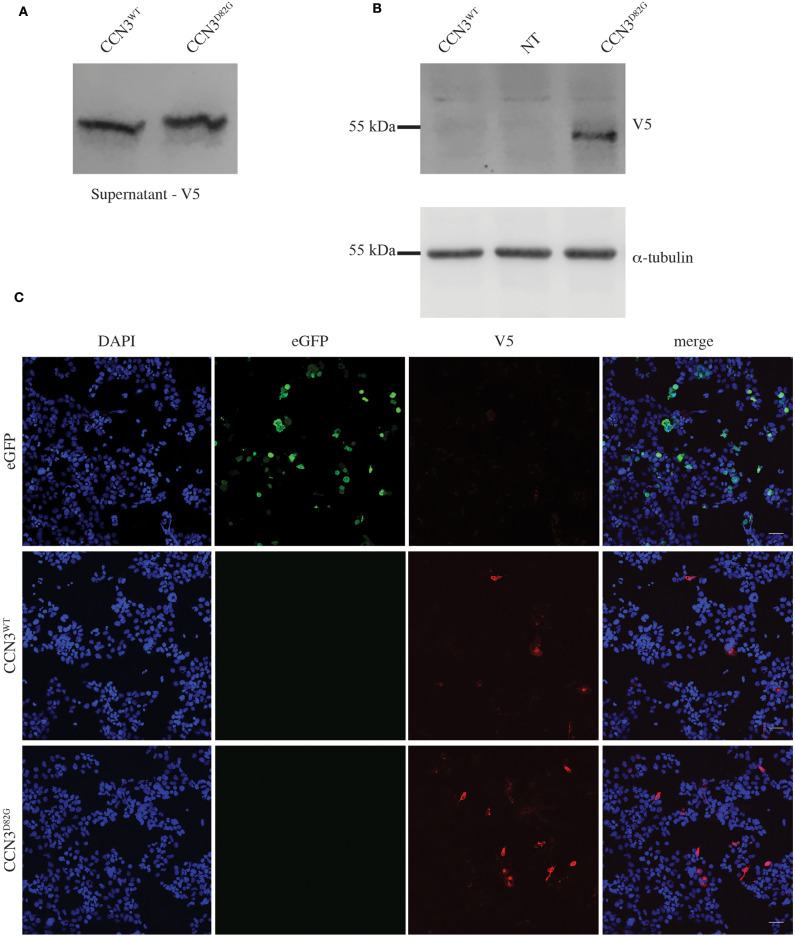
CCN3^D82G^ has impaired secretion. **(A)** V5 Immunoblot of cultured supernatant from non-transfected (NT) and CCN3^WT−^ and CCN3^D82G^-expressing cells. **(B)** Immunoblots of lysates from non- NT and CCN3^WT−^ and CCN3^D82G^-expressing cells probed with either V5 or α-tubulin. **(C)** Immunofluorescence imaging of eGFP (enhanced green fluorescent protein) and CCN3^WT−^ and CCN3^D82G^-expressing cells.

To assess if the p.D82G mutation had any effect on the CCN3 protein, both CCN3^WT^ (wild-type) and CCN3^D82G^ were expressed in HEK293T cells. Due to the fact that CCN3 is a secreted protein, CCN3^WT^ was primarily detected in the cell culture medium as expected (Figure 2A), with barely any detectable protein within the cell (Figure 2B). Furthermore, CCN3^D82G^ was also detected at a similar level in the cell culture medium (Figure 2A). However, when the CCN3^D82G^ cell lysate was analyzed, we detected a significant increase in cellular CCN3 protein (27.65 ± 13.01 fold increase, *p* <0.05; Figure 2B). We next investigated the subcellular localization of CCN3^D82G.^ by immunofluorescence staining of HEK293 cells transfected with either CCN3^WT^ or CCN3^D82G^. Subcellular localization confirmed CCN3^WT^ was detectable in less transfected cells than the CCN3^D82G^ (Figure 2C), suggesting the CCN3^D82G^ had impaired secretion when compared to CCN3^WT^.

**Figure 3:**
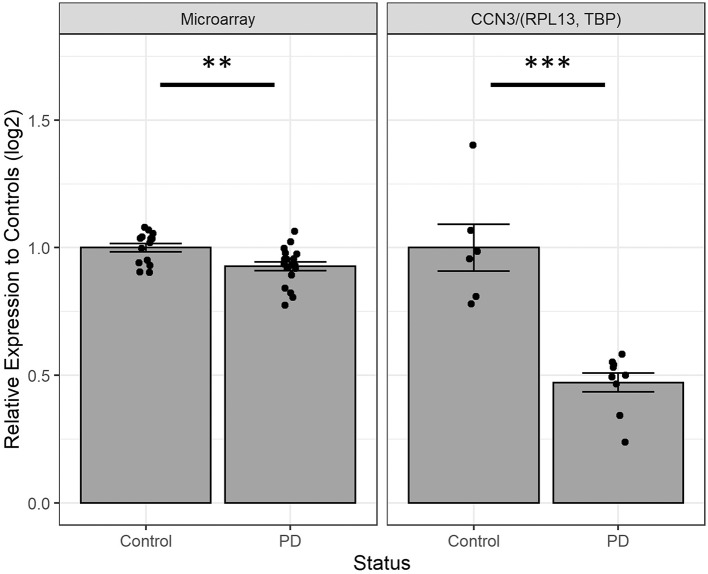
Comparing *CCN3* mRNA expression between cases and controls from microarray and qPCR data. Expression data were normalized to control-derived hONS samples and displayed on a log scale. qPCR data were normalized to geometric mean of *RPL13* and *TBP*. Error bars represent standard error. **p <0.01, ***p <0.001.

We next investigated the expression of *CCN3* mRNA in hONS cells derived from unrelated sporadic cases and controls to validate observations from microarray data (16), which suggested that *CCN3* was decreased by 7.3% in PD patients (*p* <0.01; Figure 3). Interestingly, the qPCR analysis confirmed *CCN3* expression was downregulated in PD cases by 52.8% (*p* <0.001) when compared to *RPL13* and *TBP* (Figure 3).

## Discussion

Here we present evidence that a rare homozygous mutation in *CCN3* found in a family in rural Pakistan may be a novel cause of parkinsonism. After excluding other causes of early-onset parkinsonism within affected family members, homozygosity mapping and WES identified only one suitable candidate for disease: *CCN3* c.A245G (p.D82G). Prior to this study, the mutation had only been reported once as a heterozygous variant in a European subject above 80 years of age in the gnomAD dataset (20). We did not detect the variant in 353 Australian samples, which included 57 multi-incident families and 104 early-onset cases. Further, the mutation was not detected in three other WES datasets with a combined total of 6,434 samples of which 3,390 were of Pakistani heritage. These data suggest the mutation is very rare across multiple populations; however, screening the community from the same rural district as the family may provide further insight into the local frequency of the sequence variant. Although the CCN3 aspartic acid residue at position 82 is multi-allelic, the reported rate of the asparagine and glutamic acid amino acid changes were still rare (20), have lower CADD scores compared to the glycine substitution, 24.4 and 18.2, respectively (19), and may have different effects on protein function. Notably, glycine is achiral and has been noted to affect flexibility in protein conformation (21, 22). Hence, we still consider this mutation as a good candidate for disease in affected members of this family.

Notably, we observed that a portion of the CCN3^D82G^ protein is consistently retained within the cell, while still being able to be secreted. This suggests that the aspartic acid residue in the insulin-like growth factor (IGF)-like binding domain is important for normal secretion. This finding is interesting as the CCN3 IGF-like binding domain has little to no IGF binding affinity (23), thus supporting the importance of this domain for another function.

Mechanisms in which this aberrant CCN3 protein may lead to disease are unclear. It is noteworthy that *CCN3* mRNA is expressed in developing human brains and has been detected in the substantia nigra, pontine abducens, thalamic nuclei, and striatum at 32 weeks gestation (24). Further, the CCN3 protein was observed in motor neurons of the ventral horn (25). Thus, CCN3 may have a role in the development of the nervous system. Alternatively, chemokines CCL2 and CXCL1 in rat astrocytes were found to increase upon recombinant CCN3 protein exposure (26), suggesting a possible neuroinflammatory role. Further investigation is strongly warranted to characterize potential mechanisms affected by the aberrant CCN3 protein.

It is also noteworthy that hONS cells from sporadic PD cases had a lower expression of *CCN3*. These primary human cells were previously shown to encapsulate aspects of disease, such as metabolic, oxidative (16, 27), and mitochondrial phenotypes (28). Consistently, the disease-specific decreased expression was also observed through microarray analysis (16). These data suggest *CCN3* expression may have a role in sporadic disease, or conversely, disease status may have an effect on *CCN3* expression. This observation also warrants further investigation.

## Conclusion

While we cannot exclude mutations residing outside the exome, the evidence presented in this study indicates that the best candidate for disease in this family was the rare homozygous *CCN3* p.D82G mutation. Notably, the mutation impaired secretion of the CCN3 extracellular matrix protein; however, the pathway affected by the aberrant protein within the affected family requires further investigation. We propose that other consanguineous families with early-onset parkinsonism from this region should also be examined for this and other rare variants. Interestingly, this study also presents evidence to suggest that *CCN3* expression is downregulated in idiopathic PD; however, the molecular causes of this downregulation and the role CCN3 has in idiopathic PD also require further elucidation. Nevertheless, this study has identified a strong interesting parkinsonism candidate, CCN3, highlighting the utility of screening isolated affected families for identification of putative disease-causing genes.

## Data Availability Statement

The datasets generated for this study are available on request to the corresponding author.

## Ethics Statement

The studies involving human participants were reviewed and approved by Postgraduate Medical Institute (PGMI), Lady Reading Hospital (LRH), Peshawar (Ref. 65/IRBEB/PGMI/LRH) and the Human Research Ethics Committee (HREC) at Griffith University (Ref: ESK/04/11/HREC). The patients/participants provided their written informed consent to participate in this study. Written informed consent was obtained from the individual(s) for the publication of any potentially identifiable images or data included in this article.

## Author Contributions

SK, ZA, and MN contributed to the collection of patient information and sample. SB, SK, and JF conducted the genetic analysis. SB and MÖ conducted and analyzed the genotyping. SB and SP conducted and analyzed the expression analysis. JI designed the expression construct. AS conducted and analyzed cell culture, immunoblotting, and immunostaining. GM led and supervised the overall project. SB, AS, SK, GM, SW, JF, and CL contributed to study design and conception. SB and AS prepared the manuscript. All authors contributed to the manuscript revision.

## Conflict of Interest

GM is a research topic editor. The remaining authors declare that the research was conducted in the absence of any commercial or financial relationships that could be construed as a potential conflict of interest.
